# Impact of control of blood glucose level during treatment of sudden deafness in diabetics: relationship with prognosis

**DOI:** 10.1007/s00405-016-4388-4

**Published:** 2016-11-16

**Authors:** Sang-Ki Min, Ji-Ho Shin, Mun-Young Chang, Hyun-Jin Min, Kyung-Soo Kim, Sei-Young Lee, Hoon-Shik Yang, Young-Ho Hong, Seog-Kyun Mun

**Affiliations:** Department of Otorhinolaryngology-Head and Neck Surgery, College of Medicine, Chung-Ang University Hospital, 102 Heukseok-ro, Dongjak-gu, Seoul, 06973 Korea

**Keywords:** Diabetes, Blood glucose level, Sudden deafness

## Abstract

The objective of this study is to investigate the impact of control of blood glucose level during treatment of sudden deafness. A retrospective study was performed involving 197 patients from January, 2011 to September, 2015. All patients were administrated prednisolone (Pharmaprednisolone tab^®^, 5 mg/T; KoreaPharma) p.o under the following regimen: 60 mg/day for 4 days, 40 mg/day for 2 days, 30 mg/day for 1 day, 20 mg/day for 1 day, and 10 mg/day for 2 days. During treatment, pure tone audiometry and blood glucose level were investigated for each patient and the results were statistically analyzed. Mean hearing improvement was 19.2 dB for the non-diabetes group and 24.8 dB for the diabetes group. The greater improvement for diabetics was not statistically significant (*p* = 0.146). Hearing improvement was 25.1 dB for subjects with mean blood glucose <200 mg/dl and 24.6 dB for subjects with mean blood glucose >200 mg/dl; the difference was not statistically significant (*p* = 0.267). Mean blood glucose level was 200.8 mg/dl for subjects with hearing improvement >20 dB and 181.8 mg/dl for subjects with hearing improvement <20 dB; the difference was not statistically significant (*p* = 0.286). Control of blood glucose level during treatment of sudden deafness does not have a direct effect on prognosis.

## Introduction

Sudden deafness is defined as the sudden development of unilateral or bilateral sensorineural hearing loss. Virus infection and blood circulation disorder are considered as major etiologies, but the exact cause still remains controversial [1]. Mechanisms involved in the induction of hearing loss by different viruses vary greatly, raging from damage to direct inner ear structures, to induction of host immune-mediated damage. Currently, steroid use is an effective treatment in treating patients with sudden deafness [2]. However, hyperglycemia in patients with diabetes remains as an obstacle in treating sudden deafness. Studies have investigated the prognosis of sudden deafness with diabetes as a factor [1, 2], but prognosis according to hyperglycemia during steroid treatment has not yet been studied. The purpose of our study was to suggest mechanism that blood glucose level during steroid treatment could affect improvement in sudden deafness patients.

## Materials and methods

### Subjects

The retrospective study involved 197 patients diagnosed with sudden deafness with unknown origin in our hospital from January, 2011 to September, 2015. Fifty-seven patients with type 2 diabetes were included and a thorough physical examination and systemic review were conducted for each patient. All diabetic patients had been receiving oral hypoglycemic medication such as metformin (Diabex Tab^®^, 500 mg/T; Daewoong) and glimepiride (Amaryl Tab^®^, 2 mg/T; Handok) were on regular follow-up for their blood glucose levels. The initial glucose level upon admission was fairly under control which was 143 mg/dl. For the diabetes group, they were divided into 2 groups; group I as glucose level >200 mg/dl, and group II as <200 mg/dl. The reason for this division was to investigate whether glucose levels during treatment had effect on prognosis. Also, the diabetes group was again categorized into group A as patients with hearing improvement >20 dB, and group B with hearing improvement <20 dB. This was to investigate whether patients who showed improvement in sudden deafness also had well-controlled glucose level during treatment.

Patients who commenced treatment within 7 days of onset were included but those who had vertigo, tinnitus were excluded from the study. Also, routine blood tests and serological viral studies were performed to exclude viral, inflammatory, traumatic origin. This study was determined to be exempt from review by the institutional review board at our institution.

### Treatment method

All patients were administrated prednisolone (Pharmaprednisolone tab^®^, 5 mg/T; KoreaPharma) p.o under the following regimen: 60 mg/day for 4 days, 40 mg/day for 2 days, 30 mg/day for 1 day, 20 mg/day for 1 day, and 10 mg/day for 2 days. Control of blood glucose was done through self-medication for each patient. For control of hyperglycemia, Insulin aspart (Novorapid^®^, 100 IU/ml; Novo Nordisk) was administered subcutaneously. If glucose level was greater than 200 mg/dl, 4 units of Insulin aspart was administered upon consultation with internal medicine doctors. For every 50 mg/dl increase in glucose level, 1 unit of Insulin aspart was increased. Blood glucose stick test was performed 1 h after insulin injection to follow up blood glucose levels.

### Audiometry and blood glucose level examination

Initial pure tone audiometry was performed before treatment and once every 2 days during treatment. Pure tone audiometry was performed 1 month after treatment for final evaluation. Audiometry was evaluated using four frequencies (0.5, 1, 2, and 3 kHz). Patients had their meal at 7 am, 12 pm, and 6 pm. Glucose stick test was performed four times daily (7 am, 10 am, 4 pm, and 9 pm) to measure fasting glucose. Two-hour pre-prandial glucose and 3-h post-prandial glucose tests were done. Although Hb A1c (glycosylated hemoglobin) is known as a good indicator of long term glucose control, glucose stick test was used in this study to evaluate the four specific periods each day after steroid was administered.

### Statistical analysis

All statistical evaluations were performed using SPSS version 12.0 (SPSS, Chicago, IL, USA) and *p* values <0.05 were considered significant. Comparison of hearing improvement between the diabetes group and non-diabetes group was done using Mann–Whitney *U* test and evaluation of hearing improvement according to control of blood glucose level was done using the Kruskal–Wallis test.

## Results

### Objective hearing improvement in diabetes and non-diabetes patients

**Fig. 1:**
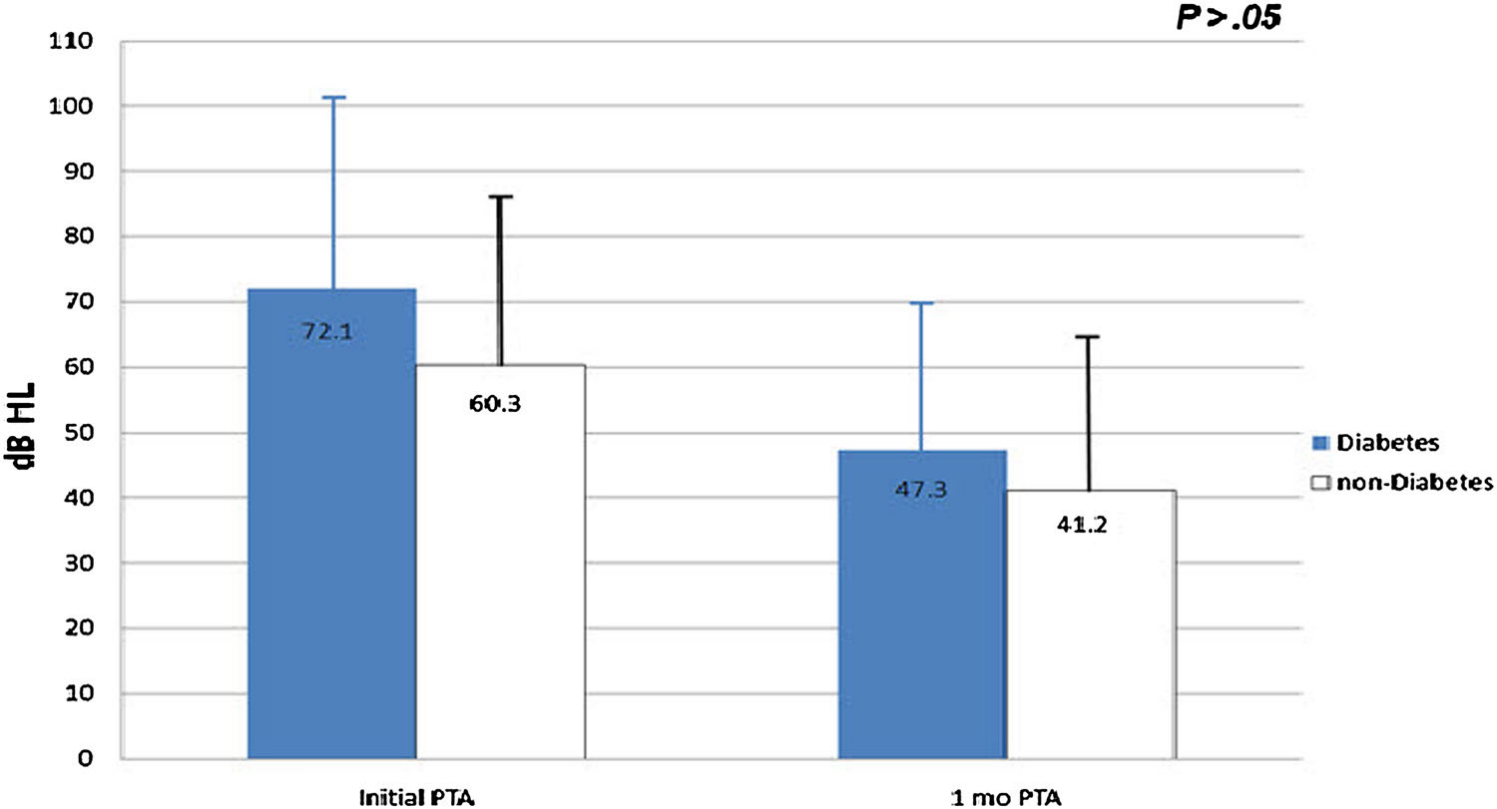
Comparison of initial and final (1 month) pure tone average between diabetes and non-diabetes group

The 197 patients included 140 non-diabetes patients in which 63 were male, 77 were female, and mean age was 46.8 years. For the non-diabetes group, the average initial pure tone audiometry was 60.3 dB and final audiometry performed 1 month after treatment was 41.2 dB. There were 57 diabetes patients, comprising 34 males, 23 females; their mean age was 57.4 years. Average initial pure tone audiometry for the diabetes group was 72.1 dB and final audiometry was 47.3 dB (Fig. 1). By defining hearing recovery as improvement in pure tone audiometry >10 dB, 76 patients (54.3%) in the non-diabetes group showed hearing recovery and 37 patients (64.9%) in the diabetes group showed hearing recovery. Hearing improvement was measured from the difference in the initial and final pure tone audiometry. Hearing improvement was 19.2 dB for the non-diabetes group and 24.8 dB for the diabetes group; the greater improvement for the diabetes group was not statistically significant (*p* = 0.146) (Table 1).

**Table 1:** Comparative data regarding diabetes and non-diabetes group

	Diabetes group (*n* = 57)	Non-diabetes group (*n* = 140)
Age (years)	57.4 ± 13.9	46.8 ± 15.8
Sex (M:F)	34:23	63:77
Hearing recovery (%)	37 (64.9)	76 (54.3)
Initial audio (dB)	72.1 ± 22.9	60.3 ± 26.8
Final audio (dB)	47.3 ± 23.1	41.2 ± 25.1
Improvement value (dB)	24.8 ± 18.7	19.2 ± 22.6

### Hearing improvement according to control of blood glucose level

**Fig. 2:**
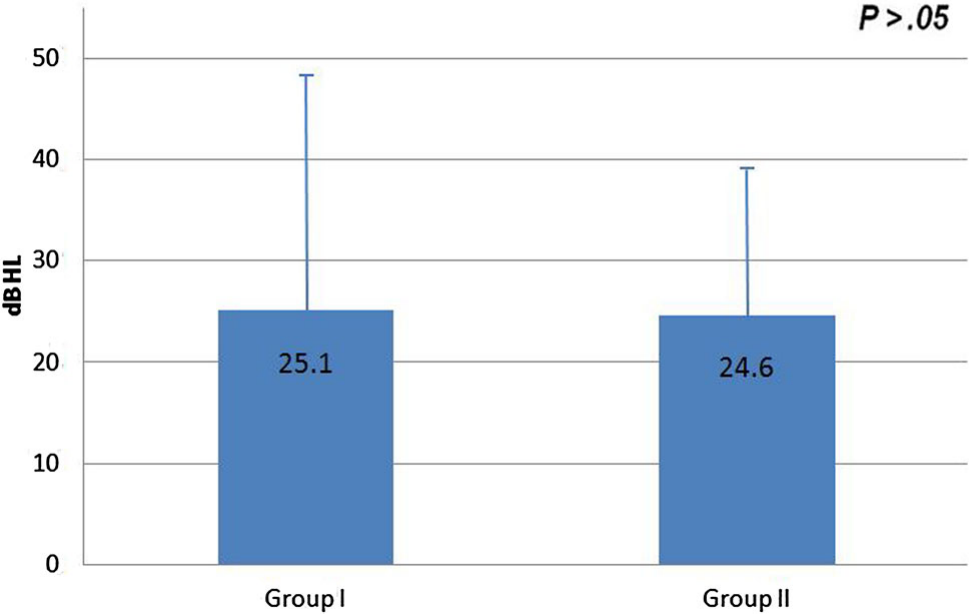
Comparison of hearing improvement in pure tone average between non-glucose level control group (Group I) and glucose control group (Group II)

Mean glucose level was obtained by the average of the glucose stick test result during treatment, and glucose level >200 mg/dl was categorized as group I, which included 24 patients, and <200 mg/dl as group II, which included 33 patients. Hearing improvement according to control of blood glucose level was 25.1 dB for group I and 24.6 dB for group II, but it was not statistically significant (*p* = 0.267) (Fig. 2).

### Comparison of blood glucose level according to hearing improvement

**Fig. 3:**
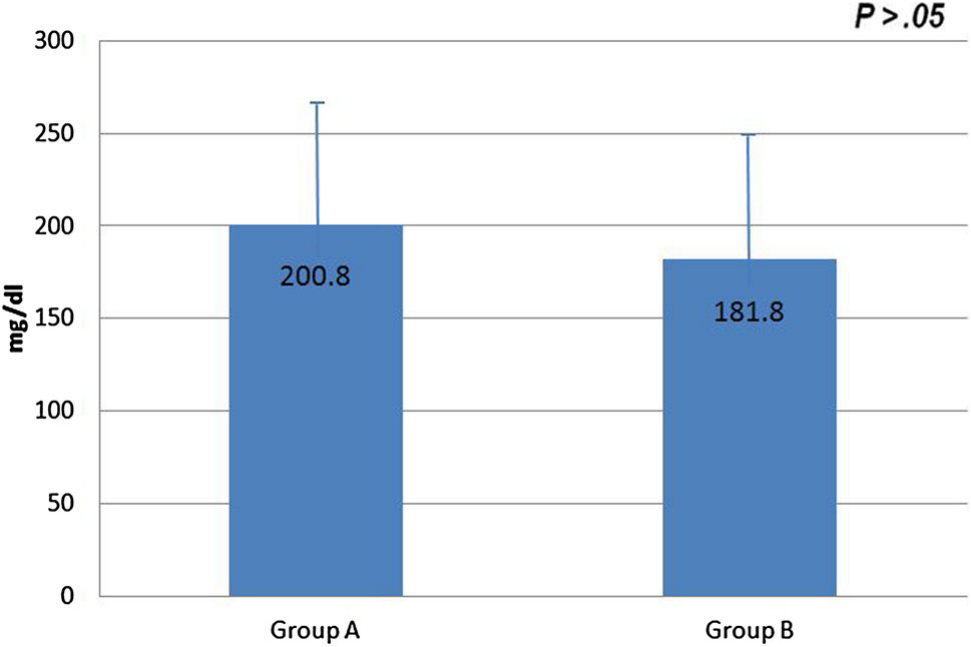
Comparison of mean blood glucose level between patients with hearing gain greater than 20 dB (Group A) and patients with hearing gain less than 20 dB (Group B)

Patients with hearing improvement >20 dB in pure tone audiometry was categorized as group A, which included 30 patients, and <20 dB as group B, which included 27 patients. The mean blood glucose level according to hearing improvement was 200.8 mg/dl for group A and 181.8 mg/dl for group B, and the difference was not statistically significant (*p* = 0.286) (Fig. 3).

## Discussion

The etiology of sudden deafness is still not clearly known. Viral infection and disorders in blood circulation are thought to be major reasons, but cochlear membrane rupture, immunodeficiency disease, acoustic tumor, and head trauma can also cause sudden deafness. The association with viral infection has been gleaned from some cases that were serologically positive, but the exact pathophysiological route or viral infection process remains unclear [3]. Also, since the cochlea is supplied by the labyrinthine artery, which has no collateral circulation, vessel obstruction by thrombosis may cause sudden deafness that cannot be identified apparently [4]. Diabetes, hyperlipidemia, and old age are well-known factors of microvessel disease, and these factors also support the theory of disorder in blood circulation [5].

Systemic steroid administration is the most popular treatment method for sudden deafness of unknown origin. High dose steroid therapy enables anti-inflammatory, immunosuppressive, and regulatory effects in vessel permeability. The therapy can prevent edema in the inner ear and also suppresses fibrogenesis or scar tissue formation, which aids in the recovery process by reducing damage in the inner ear caused by viral infection. Steroids are involved in the immunosuppression process by reducing the expression of nuclear factor-kappa beta (NF-κB), a transcription factor in the immune system. NF-κB is distributed inside the cochlea and steroid administration has been reported to reduce this transcription factor. Steroids also have a regulatory effect in vessel permeability and are involved in cochlea blood circulation by controlling homeostasis in endolymphatic fluid [6].

There is little doubt concerning the effectiveness of steroids in sudden deafness. However, high dosage therapy (prednisolone 60 mg) is reportedly linked with complications, most commonly hyperglycemia. In animal experiments, continuous retention of dexamethasone or a substance interrupting 11β-HSD2 results in hyperglycemia and hyperinsulinemia [7]. Since hyperglycemia developing during steroid therapy may result in poor prognosis, there is always a dilemma between control of blood glucose level and steroid use. Currently, intra-tympanic steroid injection is used to avoid systemic complications. However, the therapeutic outcome is variable, with reported success rates of 53 and 72.7% [8, 9]. These results indicate the similarity of outcome of the intra-tympanic method compared to traditional systemic steroid therapy. However, if the patient swallows or talks during the process, the injected medication may be lost through the Eustachian tube and may not be sufficiently absorbed through the round window. Also, if the round window niche is covered by a pseudomembrane, it may reduce steroid absorption and result in lower treatment effect compared to systemic steroid therapy.

Presently, comparison of the level of hearing improvement between diabetes patients and non-diabetes patients revealed a greater (but not statistically significant) hearing improvement in diabetes patients. This result was contrary to prior reports [2, 5, 10–12] that patients with diabetes have a poor prognosis. Also, a formulation in which prostaglandin E1 is enclosed in a lipid capsule reportedly has no statistically significant treatment effect in sudden deafness [13, 14], but this may be because microvessel disease does not easily develop in the labyrinthine artery compared to other vessels. However, the study was limited by the relatively small number of total patients and because final pure tone audiometry was performed 1 month after initial treatment, which may have resulted in a lack of statistical difference.

Sudden deafness in patients with diabetes has been linked with more severe hearing loss and poor prognosis [10, 11]. Also, the post-prandial glucose level and control of blood glucose level likely affect the course of disease [10]. However, no study has reported the impact of hyperglycemic control in the prognosis of sudden deafness in patients undergoing high dose steroid therapy. Presently, we compared the level of hearing improvement and mean blood glucose levels between two groups in which there was no statistical difference. This conclusion was evaluated in two ways. First, diabetic patients were divided into two groups, in which one group showed high glucose levels and the other group showed normal blood glucose levels. Results showed that there was no statistical difference in hearing improvement between the two groups. Second, the diabetic patients were again categorized, in which one group showed hearing improvement >20 dB and the other group showed hearing improvement <20 dB. Again, there was no statistical difference in mean blood glucose levels between the two groups. Hence, from these studies, we were able to conclude that even though sudden deafness associated with diabetes is known to have a poor prognosis, our study showed that there was no correlation between the two diseases.

Also, there were some patients who complained of headaches and mild edema of the limbs, but there were no patients who developed severe symptoms such as hyperglycemic hyperosmolar nonketotic syndrome. All patients were well controlled with conservative treatment. Therefore, all patients received the 10-day regimen treatment without interruption and this was probably due to the frequent glucose stick test and the swift intervention for glucose level control using insulin injection.

A change in blood glucose level developing during systemic therapy in sudden deafness patients is a normal response and may be worsened in patients with diabetes. However, this has no direct effect on the prognosis of sudden deafness; so there should be no restrictions to systemic steroid therapy due to hyperglycemia.

## Conclusion

There is still no clear reason for the etiology of sudden deafness, but steroid therapy is currently the most popular treatment method. Sudden deafness in diabetic patients has been associated with poor prognosis in prior studies, but not in the present study. This may be due to other factors causing sudden deafness other than disorders in blood circulation. Also, since hyperglycemia is the most common complication of steroid treatment, a balance between steroid usage and hyperglycemia in patients with diabetes is difficult. Blood glucose level needs to be controlled, since hyperglycemia may cause many complications including tissue damage of the endocrine system. However, systemic steroid therapy should be administered without restrictions since control of blood glucose level during treatment do not have a direct effect on the prognosis of sudden deafness.
